# The effects of leaf litter nutrient pulses on *Alliaria petiolata* performance

**DOI:** 10.7717/peerj.1166

**Published:** 2015-08-20

**Authors:** Robert W. Heckman, David E. Carr

**Affiliations:** 1 Department of Environmental Sciences, University of Virginia, Charlottesville, VA, USA; 2 Blandy Experimental Farm, University of Virginia, Boyce, USA; 3 Current affiliation: Department of Biology, University of North Carolina, Chapel Hill, NC, USA

**Keywords:** Deciduous forest understory, Garlic mustard, Phenology, Nutrient release, Invasive plant

## Abstract

Nutrient pulses can facilitate species establishment and spread in new habitats, particularly when one species more effectively uses that nutrient pulse. Biological differences in nutrient acquisition between native and exotic species may facilitate invasions into a variety of habitats including deciduous forest understories. *Alliaria petiolata* (Bieb.) Cavara & Grande is an important invader of deciduous forest understories throughout much of North America. These understory communities contain many species which perform the majority of their growth and reproduction before canopy closure in spring. Because *A. petiolata* is a wintergreen biennial that can be active during autumn and winter, it may utilize nutrients released from decaying leaf litter before its competitors. To investigate this we manipulated the timing of leaf litter addition (fall or spring) and experimentally simulated the nutrient pulse from decaying leaves using artificial fertilizer. To determine whether *A. petiolata* affected the abundance of understory competitors, we also removed *A. petiolata* from one treatment. *A. petiolata* that received early nutrients exhibited greater growth. Treatments receiving fall leaf litter or artificial nutrients had greater *A. petiolata* adult biomass than plots receiving spring nutrient additions (leaf litter or artificial nutrients). However, fall leaf litter addition had no effect on the richness of competitor species. Thus, wintergreen phenology may contribute to the spread of *A. petiolata* through deciduous forest understories, but may not explain community-level impacts of *A. petiolata* in deciduous forests.

## Introduction

Invasive species pose a serious threat to biodiversity worldwide and have large economic and environmental costs (Mack et al., 2000; Pimentel, Zuniga & Morrison, 2005). Thus, it is important to understand the conditions that facilitate the establishment and spread of invasive species. Invasions frequently occur when a nutrient pulse increases resource availability (Huenneke et al., 1990; Davis, Grime & Thompson, 2000; Olson & Blicker, 2003; Morghan & Rice, 2006), often by one of two mechanisms: a decrease in resource uptake, or an increased supply of resources, such as a seasonal pulse from dead biomass decomposition (Davis, Grime & Thompson, 2000). Species that are able to acquire these resources before their competitors could become dominant.

In many terrestrial ecosystems, leaf litter decay is an important source of previously unavailable nutrients (Sayer, 2006; Klotzbucher et al., 2012). The cycling of nutrients from leaf litter can have important implications for these systems (Staelens et al., 2011; Klotzbucher et al., 2012). Long-term litter removal can deplete soil nutrients, especially in plant-available forms (Sayer, 2006; Leff et al., 2012), or cause strong fluctuations in soil nutrient availability (Mo et al., 2003). Nutrient release from decomposing leaf litter may enhance seedling growth (Brearley, Press & Scholes, 2003) and increase seedling survival (Sayer, 2006). Leaf litter differentially affects seedling success—suppressing shade-intolerant species while having little effect on shade-tolerant seedlings (Sydes & Grime, 1981). Although there is ample evidence for the importance of leaf litter in nutrient cycling and plant productivity (e.g., Sayer, 2006), few studies have examined the effects of leaf litter on species invasions. Most of these studies have focused on leaf litter as a physical barrier to germination and establishment (Meekins & McCarthy, 2001; Bartuszevige, Hrenko & Gorchov, 2007), yet nutrient release from leaf litter could increase the success of established invasive species (Belote & Jones, 2009).

Nutrient release and uptake are not always coupled temporally (Muller & Bormann, 1976; Anderson & Eickmeier, 2000), allowing species that are active when nutrients become available to exploit the pulse (James, Aanderud & Richards, 2006). Nutrient uptake in temperate ecosystems varies seasonally, with little winter resource utilization (Nadelhoffer, Aber & Melillo, 1984; Judd, Likens & Groffman, 2007). Thus, nutrients released during this period may remain unused for long periods (Nadelhoffer, Aber & Melillo, 1984). Wintergreen plants—herbaceous species that retain their leaves during the winter—may be able to use these nutrient pulses earlier than other species.

*Alliaria petiolata* (M. Bieb) Cavara & Grande is a wintergreen biennial in North America (Cavers, Heagy & Kokron, 1979; Rodgers, Stinson & Finzi, 2008), and one of the most successful invaders of deciduous forests throughout eastern North America (Welk, Schubert & Hoffmann, 2002; Rodgers, Stinson & Finzi, 2008; Knight et al., 2009). *A. petiolata* can reduce diversity and inhibit seedling regeneration in forest understories (Stinson et al., 2006; Stinson et al., 2007; Rodgers, Stinson & Finzi, 2008). Although it is less active during the winter, growth does occur on warmer winter days (Myers, Anderson & Byers, 2005). The relatively high light that *A. petiolata* receives during winter may allow it to bolt early in spring (Myers, Anderson & Byers, 2005). Additionally, *A. petiolata* increases its growth rate quickly in response to increased *N* availability (Hewins & Hyatt, 2010). Nutrients acquired during winter may facilitate this rapid spring growth, particularly because light availability is high before overstory trees leaf out (Augspurger, Cheeseman & Salk, 2005; Augspurger, 2008). In this study we investigated whether early nutrient availability increased adult *A. petiolata* size and fecundity. We predicted that plants receiving early (fall) nutrients would be larger and have higher reproductive output than plants receiving either late (spring) nutrients or no nutrient inputs. Furthermore, we predicted that plants receiving late nutrient inputs would be larger and have higher reproductive output than plants receiving no nutrient inputs. We also predicted that receiving early nutrients would increase *A. petiolata*’s advantage over competitor species, reducing competitor species richness and abundance.

## Materials and Methods

### *Alliaria petiolata*

*A. petiolata* is an invasive understory plant in the family Brassicaceae that is native to Eurasian temperate forests and is an obligate biennial in North America (Cavers, Heagy & Kokron, 1979; Dhillion & Anderson, 1999). *A. petiolata* was first recorded in North America in 1868 on Long Island, New York (Nuzzo, 1991). In Virginia, seedlings germinate in March and form a non-reproductive rosette consisting of several small leaves. The plant overwinters as a rosette, growing slowly until early spring when stems elongate rapidly with each individual shoot producing numerous small flowers, each of which produces a silique (Cavers, Heagy & Kokron, 1979). Silique maturation and seed set occurs primarily in June and plants senesce by late summer. Flowers are usually insect pollinated but can be self-pollinated (Anderson, Dhillion & Kelley, 1996; Cruden, McClain & Shrivastava, 1996). Seeds can remain dormant in the seedbank for up to five years (Baskin & Baskin, 1992), allowing *A. petiolata* to quickly exploit disturbances, especially along forest edges and flood plains (Nuzzo, 1999; Meekins & McCarthy, 2000; Stinson et al., 2006). Allelopathic root exudates may give *A. petiolata* an advantage over native species (Roberts & Anderson, 2001; Callaway et al., 2008).

### Study site

The 17-hectare, second growth woodlot at Blandy Experimental Farm in Boyce, Virginia, USA (39°05′N, 78°03′W) had a well-established population of *A. petiolata*. This 100-year old forest had a canopy dominated by deciduous species, mainly *Carya tomentosa*, *Celtis occidentalis*, *Quercus alba*, *Quercus rubra*, *Nyssa sylvatica*, *Prunus serotina* and *Liriodendron tulipifera*. The shrub layer consisted predominantly of *Cornus florida*, *Asimina triloba*, *Viburnum prunifolium*, *Sassafras albidum* and *Lindera benzoin*. Several exotic species, including *Lonicera maackii* and *Ligustrum* spp., were also abundant. The herbaceous understory contained many perennial spring ephemeral species that perform most of their growth and reproduction between snowmelt and canopy closure (Rothstein, 2000). These spring ephemerals may be affected by shading from *A. petiolata*—which can reach 1.25 m in height (Cavers, Heagy & Kokron, 1979)—during this time of otherwise high light to the forest understory.

### Nutrient manipulation

On 22 October 2006 we established 90 plots, each 0.5 m × 0.94 m, in a natural *A. petiolata* population. Naturally established *A. petiolata* rosette density ranged from 8 to 76 rosettes m^−2^ within the plots. We surrounded each plot with a 15 cm high barrier using landscaping fabric (2 oz/yd^2^, Greenscapes General Purpose Landscape Fabric, Calhoun, Georgia, USA), to prevent leaves and other debris from blowing onto the plot. Each plot was covered by a mesh net 60 cm above the ground in order to catch falling leaves. We cleared nets weekly to prevent shading and leaching of nutrients from debris and removed nets after leaf fall had ceased in late November. Plots were arranged in 15 spatial blocks, and each of the six treatments described below was randomly assigned to one plot in each block.

The experiment comprised six treatments. Five of these varied in the type and timing of nutrient addition: fall leaves (FL), fall artificial nutrients (FAN), spring leaves (SL), spring artificial nutrients (SAN), no inputs (NI) (Table 1). The sixth treatment (RM) was used as a control to determine the effects of *A. petiolata* on the abundance and diversity of other understory species. In RM plots we removed all *A. petiolata* rosettes. Forest floor leaf litter was left undisturbed in FL and RM plots, and litter caught above these plots was distributed evenly across the plot. Supplemental litter was added as required to match the leaf litter depth immediately outside the plot (typically 2.5–5 cm in depth) and was consistent among plots within a spatial block. Replicates of the four treatments that did not receive autumn leaf litter (FAN, SL, SAN and NI) were cleared of leaf litter beginning in October and continuing until leaf fall ceased in late November 2006. In FAN and SAN plots, artificial fertilizer was used to mimic the natural loss of nutrients from leaf litter decomposition and leaching. Artificial nutrients were added to plots to isolate the effects of nutrient addition from other properties of leaf litter that may positively or negatively affect plant growth (e.g., mutualistic or pathogenic bacteria or fungi associated with leaf litter). These nutrients were provided from slow release Osmocote^®^ fertilizer (13-13-13 NPK, Scotts Company, Marysville, Ohio, USA) at 0.994 g per plot on FAN and SAN plots on 19 November 2006 and 24 March 2007, respectively. This nitrogen addition was similar to the amount (0.283 g N m^−2^ yr^−1^) released from leaf litter in a mixed deciduous forest (Cromack & Monk, 1974). Litter that we collected in the fall 2006 and stored dry over the winter was applied to SL plots on 24 March 2007. NI plots were maintained without leaf litter or nutrient addition throughout the experiment. For each treatment in which leaves were removed (FAN, SAN, SL, and NI), we introduced artificial leaves into the plots in November 2006 to mimic physical properties of litter that may have had both positive (e.g., moisture retention and insulation) and negative (e.g., shading) effects on plant growth. We added ∼20 of these artificial leaves (∼20 × 20 cm squares), cut from landscaping fabric (Greenscapes General Purpose Landscape Fabric) to each plot. As with natural leaves, these artificial leaves were placed haphazardly within each plot. We carefully removed most artificial leaves from SAN plots prior to fertilization, however some leaves were left in place to avoid disturbing the vegetation.

**Table 1:** *A. petiolata* treatment manipulations.

Treatment	Code	Description
Fall Leaves	FL	Leaves collected in screen spread evenly over plot.
Fall Artificial Nutrients	FAN	Leaves discarded. Ground clear, Fall artificial nutrient addition.
Spring Leaves	SL	Leaves collected in autumn, reapplied to plot in spring.
Spring Artificial Nutrients	SAN	Leaves discarded. Ground clear, spring artificial nutrient addition.
No Inputs	NI	Leaves collected and discarded. Ground free of debris.
*A. petiolata* Removal	RM	*A. petiolata* removed. Leaves collected, spread on plot.

Between our fall and spring nutrient additions, the mean high temperature was 10 °C and the mean low temperature was −2 °C. Over this 125 day period, the daily high temperature was above 0 °C nearly every day (113 times) and above 10 °C nearly half of the days (60 times). Blandy received 55 cm of snow during this period, most of which occurred in small events that melted rapidly and did not cover *A. petiolata* for long periods. Thus, 60 or more of the days between our fall and spring nutrient additions were likely conducive to at least minimal *A. petiolata* growth.

### Initial measurements

We calculated *A. petiolata* rosette density on 28 and 29 October 2006, prior to any manipulations. These served as base measurements from which to compare individuals after manipulations.

### Adult growth and reproduction

In June 2007 we harvested and separated plants into above-ground (vegetative) and reproductive biomass. Above-ground biomass was oven dried and weighed. *A. petiolata* plant density and above-ground biomass per plot were calculated. We harvested siliques from adult plants to quantify per capita and per plot silique production.

### Competitor richness

To determine the impact of *A. petiolata* on its competitors we identified all herbaceous species in each plot (Table S1). We quantified species richness and the number of individuals of all species in each plot.

### Statistical analyses

We tested all data for normality and homogeneity of variances. Competitor stem density was square root transformed to meet these assumptions. We performed repeated measures ANOVAs using PROC MIXED in SAS (SAS Institute Inc. Cary, NC Version 9.2) on *A. petiolata* plant density in October and June with nutrient treatment and time as fixed effects and block as a random effect. Autumn *A. petiolata* density (October, 2006) differed significantly among treatments (*P* = 0.031), although pairwise comparisons performed with Ryan’s *Q* failed to detect significant differences among any pairs of treatments. Nonetheless, we retained initial density as a covariate for subsequent analyses. We analyzed above-ground biomass and siliques per plot with ANCOVAs using SAS 9.2 PROC GLM with treatment as a fixed effect, block as a random effect and October *A. petiolata* density as a covariate. Plot above-ground biomass and siliques per plot were the only response variables for which the covariate effect had significant explanatory value. Because initial density did not contribute significantly to adult plant density or per capita silique production (i.e., ANCOVA was not appropriate), we performed two-way ANOVAs in SAS 9.2 using a general linear model with block as a random effect and nutrient treatment as a fixed effect on garlic mustard density, per capita silique production, and competitor species richness and plant abundance.

We performed five planned contrasts for all responses examining *A. petiolata* performance that produced a significant treatment effect: Fall leaves vs. Spring leaves; Fall artificial nutrients vs. Spring artificial nutrients; Fall nutrients (FAN and FL) vs. no inputs; Spring nutrients (SAN and SL) vs. no inputs; Fall nutrients (FAN and FL) vs. Spring nutrients (SAN and SL). We maintained a 5% experiment-wise error rate in each analysis with the Dunn-Sidak adjustment (5 contrasts, *α*′ = 0.01021). In addition to these five contrasts, we performed a sixth contrast for competitor analyses: *A. petiolata* removal (RM) vs. all other plots. Thus, the adjusted *α*′ for all competitor contrasts (6 contrasts) was 0.00851. When no contrasts showed significant differences among groups for a given response variable (e.g., silique production per plot), we performed all pairwise comparisons using the Tukey-Kramer test due to unequal sample sizes (data were lost for treatments FL and SL in Block 12).

## Results

### Above-ground biomass

**Figure 1:**
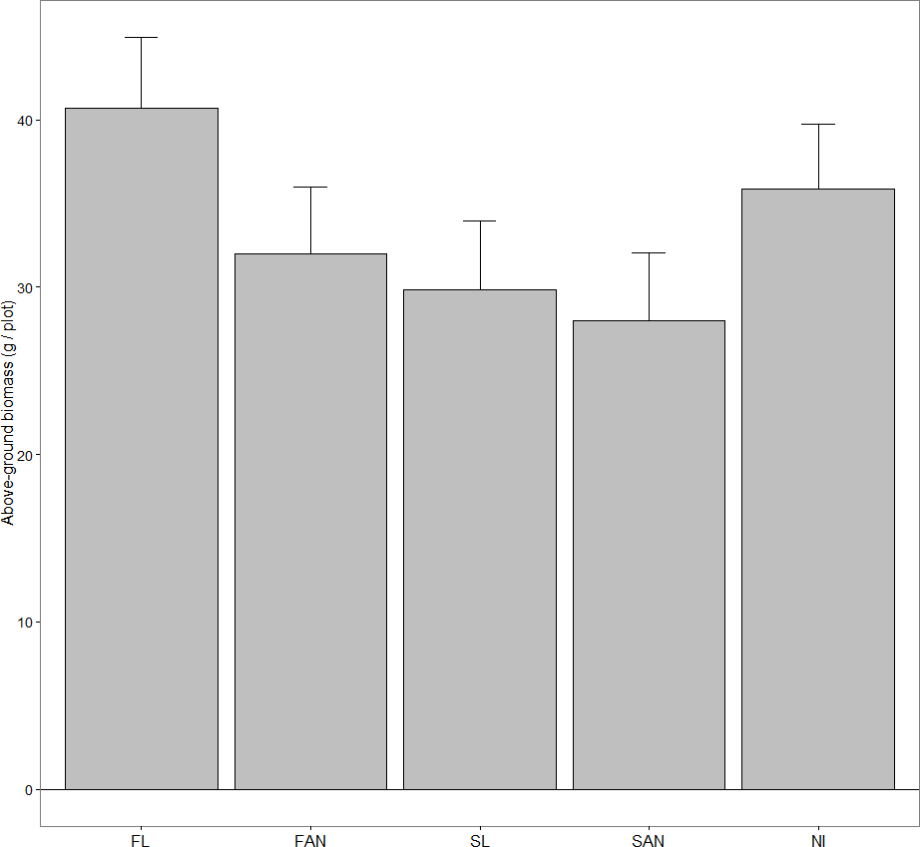
Above-ground biomass of *A. petiolata* (mean + 95% CI) harvested after seed maturation in June 2007. Treatments included: FL, fall leaves; FAN, fall artificial nutrients; SL, spring leaves; SAN, spring artificial nutrients; NI, no inputs. We performed five planned comparisons with alpha adjusted using the Dunn-Sidak method (*α*′ = 0.01274): FL differed significantly from SL; NI differed significantly from SAN + SL; FAN + FL differed significantly from SAN + SL; FAN did not differ significantly from SAN; NI did not differ significantly from FAN + FL.

There was a highly significant difference among treatments in total above-ground biomass of *A. petiolata* at senescence in June 2007 (Table 2A) when initial density was included as a covariate (*P* < 0.001). Treatment FL produced significantly more above-ground biomass than SL (Fig. 1). Plants that received fall nutrients (FL and FAN) produced significantly greater above-ground biomass than plots that received either spring nutrient input (SL and SAN). Surprisingly, plots receiving spring nutrient additions had significantly lower *A. petiolata* biomass than plots that never received nutrients (NI). Total *A. petiolata* density was not significantly different among treatments (Table 3A) when plots were harvested in June (*P* = 0.39).

**Table 2:** Results of analysis of covariance. (A) Adult *A. petiolata* above-ground biomass, (B) Silique production per plot. October juvenile rosette density was used as a covariate.

Source	(A) Biomass			(B) Siliques	
	df	MS	*F*	df	MS	*F*
Treatment	4	323.08	5.74^*^	4	56,705.99	3.45^*^
Covariate	1	1,195.13	21.23^*^	1	506,256.62	30.81^*^
Block	14	448.92	7.97	14	101,404.99	6.17
Error	53	56.31	–	53	16,431.70	–

**Notes.**

* Denotes significant effect at *P* = 0.05.

**Table 3:** Results of analysis of variance. (A) Adult plants per plot at senescence in June, (B) Silique production per adult plant.

Source	(A) Adult plant density			(B) Siliques/Plant		
	df	MS	*F*	df	MS	*F*
Treatment	4	50.42	1.05	4	304.72	3.77^*^
Block	14	158.26	3.29	14	351.95	4.36
Error	54	48.12	–	54	80.81	–

**Notes.**

* Denotes significant treatment effect at *P* = 0.05.

### Reproductive output

**Figure 2:**
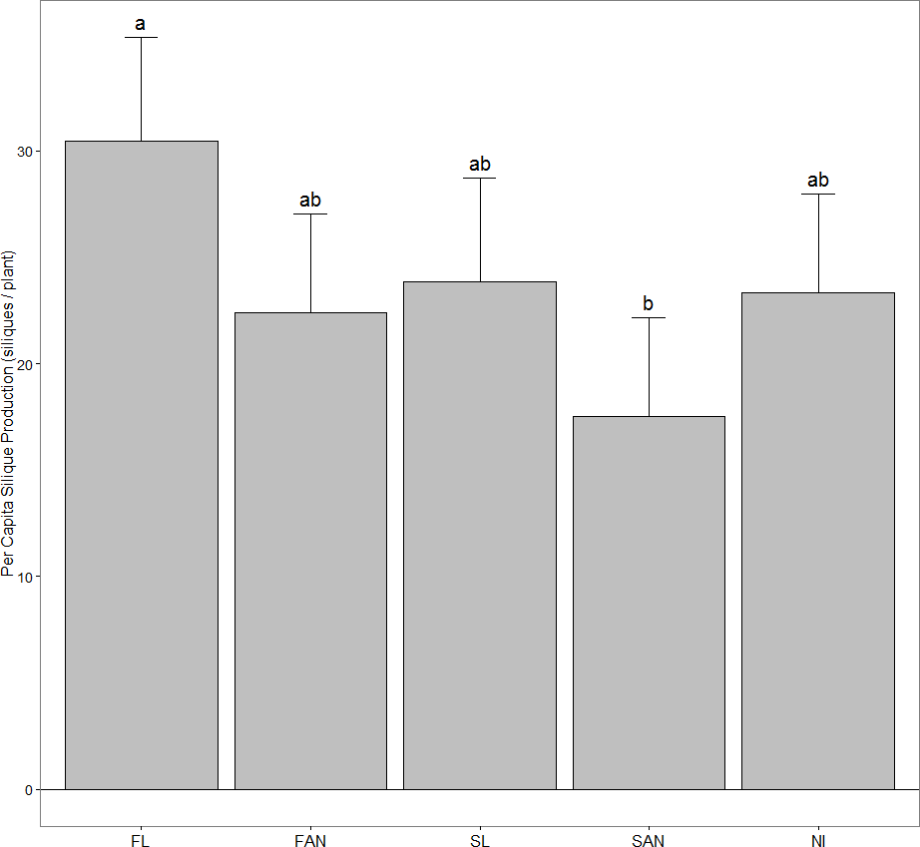
Per capita silique production in *A. petiolata* (mean + 95% CI) harvested in June 2007. Treatments included: FL, fall leaves; FAN, fall artificial nutrients; SL, spring leaves; SAN, spring artificial nutrients; NI, no inputs. Plots receiving fall leaf litter had significantly greater silique output per plant than spring artificial nutrient treatment. Shared letters denote no significant difference at *α* = 0.05. Pairwise comparisons were performed using the Tukey-Kramer method.

**Figure 3:**
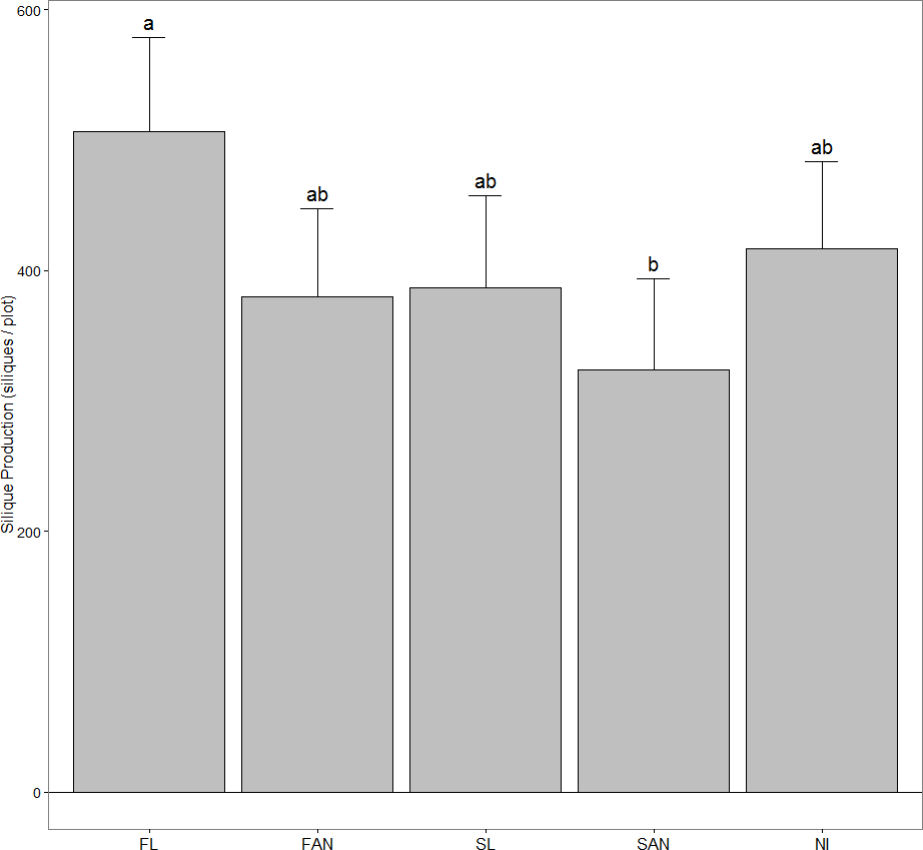
The number of *A. petiolata* siliques produced within a plot in June 2007 (mean + 95% CI). Treatments included: FL, fall leaves; FAN, fall artificial nutrients; SL, spring leaves; SAN, spring artificial nutrients; NI, no inputs. Although the overall ANCOVA showed a significant treatment effect, no contrasts produced significant differences among treatments. Shared letters denote no significant difference at *α* = 0.05. Pairwise comparisons were performed using the Tukey-Kramer method.

Plots receiving FL had significantly higher per capita silique production than SAN plots (FL, 30.45 siliques/plant; SAN, 17.50 siliques/plant) (*P* = 0.009), while SL, FAN and NI plots had approximately equal per capita silique production (Fig. 2). There was a significant treatment effect on silique production per plot when treating initial density as a covariate (*P* = 0.014) (Table 2B), however none of the planned contrasts showed significant differences among groups (Fig. 3). When examining all pairwise comparisons, FL plots produced significantly more siliques than SAN plots (FL, 510.4 siliques/plot; SAN, 328.1 siliques/plot).

### Competitor analyses

**Figure 4:**
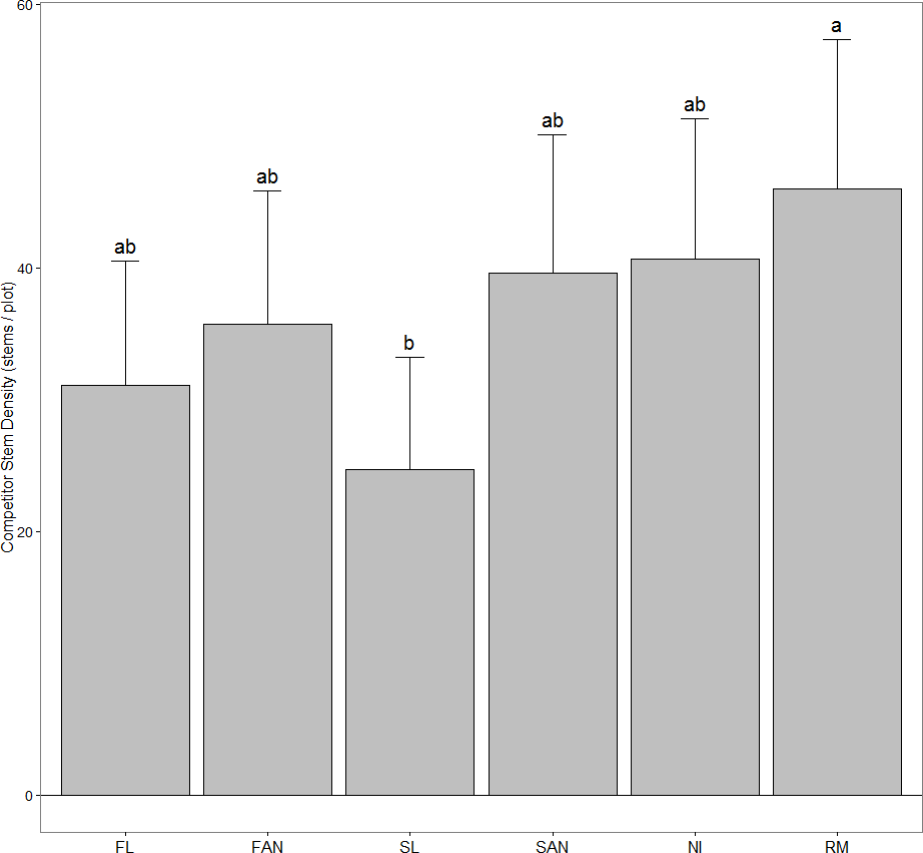
The total abundance (number of stems) of *A. petiolata*’s competitors in each plot (mean + 95% CI). Treatments included: FL, fall leaves; FAN, fall artificial nutrients; SL, spring leaves; SAN, spring artificial nutrients; NI, no inputs; RM, *A. petiolata* removed. Shared letters denote no significant difference at *α* = 0.05.

We found no treatment effects on competitor species richness in this study. However, competitor abundance (stem density, Fig. 4) was significantly affected by *A. petiolata* treatment (Table 4); plots from which *A. petiolata* was removed contained a significantly greater number of stems of competitor species (45.97) than plots receiving spring leaf-litter (24.70). There were no significant differences among any other treatments.

**Table 4:** Results of analysis of variance. (A) Richness of understory competitor species, (B) Total number of individuals of all competitor species within a plot.

Source	(A) Species richness			(B) Competitor density		
	df	MS	*F*	df	MS	*F*
Treatment	5	1.20	0.70	5	6.22	2.64^*^
Block	14	9.15	5.32	14	41.39	17.59
Error	70	1.72	–	70	2.35	–

**Notes.**

* Denotes significant treatment effect at *P* = 0.05.

## Discussion

We found some support for the hypothesis that autumn leaf litter acts as a nutrient pulse that could facilitate *A. petiolata* invasion. In this study, *A. petiolata* responded to fall nutrient additions (FL and FAN) with greater biomass production than when receiving spring nutrients. Plots that received no nutrients had greater above-ground biomass than those that received spring nutrient pulses. These results indicate that early acquisition of nutrients provided the greatest benefit to *A. petiolata*. This benefit may be enhanced by the high light availability to the understory in early spring (Myers & Anderson, 2003; Myers, Anderson & Byers, 2005). In fact, late acquisition of nutrients appears to have inhibited biomass production, perhaps due to competition for nutrients during spring with other species including trees and larger shrubs. Leger et al. (2007) found that native species in unfertilized plots were more successful than native species in fertilized plots through a combination of competition with invasive species and increased herbivory on more palatable (higher tissue *N*) plants.

Annual fecundity is an important demographic rate for *A. petiolata* populations (Davis et al., 2006). Populations that produce fewer seeds spread less rapidly and are more easily controlled than more fecund populations (Pardini et al., 2009). Sparse satellite populations should benefit more from increased silique production per individual plant, shortening the time from initial establishment to dominance (Pardini et al., 2009); furthermore, increases in total silique production per area may have a greater impact on the rate of *A. petiolata* spread and community dominance (Davis et al., 2006). Despite the importance of reproductive output in population spread, no biologically meaningful differences in *A. petiolata* silique production were detected from planned contrasts.

Variation among treatments in the initial density of *A. petiolata* rosettes may have obscured some differences among treatments in final abundance and reproductive measures in our experimental plots. The plots to which the SAN treatment was applied had higher initial density of *A. petiolata* than other treatments. We used initial density as a covariate in the analyses, but treatment effects could have been obscured if density effects were nonlinear—especially if very high densities began to suppress individual growth. In a similar experiment, extremely high density of *A. petiolata* suppressed per capita biomass, reproduction, and response to fertilization (Meekins & McCarthy, 2000). Likewise, biomass and per capita reproduction increased in experimentally thinned populations in another study (Rebek & O’Neil, 2006). However, *A. petiolata* densities in these studies were 2–3 times the typical density in our plots, so it is unlikely that substantial density-dependent reductions in per capita biomass or reproduction occurred in our study.

*A. petiolata* did not affect understory richness in this study. Although we chose areas with moderately dense *A. petiolata* populations, any declines in species richness attributable to *A. petiolata* likely occurred decades ago. Furthermore, removing *A. petiolata* from small plots would not have opened enough space for species most affected by competition with *A. petiolata* to reestablish. *A. petiolata* removal modestly increased competitor abundance, supporting the results of another study (Carlson & Gorchov, 2004). *A. petiolata* removal may, however, increase competitor richness over longer time scales than this short-term experiment (Meekins & McCarthy, 1999), although *A. petiolata* removal did not increase competitor richness in other studies (Hochstedler et al., 2007; Davis et al., 2014).

## Conclusions

Our results indicated that *A. petiolata* was capable of using fall nutrient inputs to increase growth. Relative to spring inputs or no inputs, fall nutrient inputs increased biomass. Although these results suggest that early acquisition of nutrients benefited *A. petiolata*, we were unable to detect negative effects of nutrient addition on other understory species. These nutrients benefited *A. petiolata* without directly increasing its impact on competitors. Thus, although wintergreen phenology may have facilitated the establishment of *A. petiolata* in North American forests, it appeared insufficient to explain the community-level effects of *A. petiolata* in deciduous forest understories. It is critical to more fully understand the implications for communities invaded by species possessing unique life-history traits because these traits may facilitate establishment and spread.

## Supplemental Information

10.7717/peerj.1166/supp-1 Table S1 Common competitors of *A. petiolata* Nativity, life history, and growth habit of all species occurring in more than 5 plots.

10.7717/peerj.1166/supp-2 Data S1 Heckman and Carr Supplementary data Data included in the primary dataset for all analyses, collected between October 2006 and June 2007. Mass is *A. petiolata* above-ground vegetative biomass (g) harvested from each plot in June 2007. Siliques are total number of *A. petiolata* siliques produced in each plot. Initial.Plants is the total number of *A. petiolata* individuals in each plot when treatments were applied in October 2006. Final.Plants is the total number of *A. petiolata* individuals in each plot at harvest in June 2007. Richness is the number of herbaceous species in each plot, not including *A. petiolata*. Abundance is the total number of individuals of herbaceous species in each plot, not including *A. petiolata*.
