# Supplementary material for: The effects of leaf litter nutrient pulses on Alliaria petiolata performance
Source: PeerJ. 2015 Aug 20;3:e1166. doi: 10.7717/peerj.1166 (PMC4548537; doi:10.7717/peerj.1166)
Supplement: Table S1 — Nativity, life history, and growth habit of all species occurring in more than 5 plots. [file peerj-03-1166-s001.docx]

| Species ID | Nativity | Invasive in Virginia? | Family | Life History | Growth Habit | Plots Observed |
| --- | --- | --- | --- | --- | --- | --- |
| *Duchesnea indica* | Introduced | No | Rosaceae | Perennial | Herbaceous forb | 11 |
| *Galium aparine* | Native | No | Rubiaceae | Annual | Herbaceous forb | 14 |
| *Ligustrum sinense* | Introduced | Yes | Oleaceae | Perennial | Woody | 38 |
| *Lindera benzoin* | Native | No | Lauraceae | Perennial | Woody | 8 |
| *Oxalis* sp. | Unknown | No | Oxalidaceae | Unknown | Herbaceous forb | 6 |
| *Phryma leptostachya* | Native | No | Verbenaceae | Perennial | Herbaceous forb | 9 |
| *Pilea pumila* | Native | No | Urticaceae | Annual | Herbaceous forb | 17 |
| *Polygonum cespitosum* | Introduced | No | Polygonaceae | Annual | Herbaceous forb | 66 |
| *Stellaria media* | Introduced | Yes | Caryophyllaceae | Annual/Perennial | Herbaceous forb | 47 |
| Unknown grass | Unknown | No | Poaceae | Unknown | Graminoid | 24 |
